# Transcranial Focused Ultrasound Targeting the Amygdala May Increase Psychophysiological and Subjective Negative Emotional Reactivity in Healthy Older Adults

**DOI:** 10.1016/j.bpsgos.2024.100342

**Published:** 2024-06-05

**Authors:** Bianca Hoang-Dang, Sabrina E. Halavi, Natalie M. Rotstein, Norman M. Spivak, Nolan H. Dang, Luka Cvijanovic, Sonja H. Hiller, Mauricio Vallejo-Martelo, Benjamin M. Rosenberg, Andrew Swenson, Sergio Becerra, Michael Sun, Malina E. Revett, David Kronemyer, Rustin Berlow, Michelle G. Craske, Nanthia Suthana, Martin M. Monti, Tomislav D. Zbozinek, Susan Y. Bookheimer, Taylor P. Kuhn

**Affiliations:** a Department of Psychiatry and Behavioral Sciences, University of California, Los Angeles, Los Angeles, California; b Department of Neurosurgery, University of California, Los Angeles, Los Angeles, California; c UCLA David Geffen School of Medicine Medical Scientist Training Program, University of California, Los Angeles, Los Angeles, California; d Department of Radiology, University of Colorado School of Medicine, Aurora, Colorado; e Neuroscience Interdepartmental Program, University of California, Los Angeles, Los Angeles, California; f Department of Bioengineering, University of California, Los Angeles, Los Angeles, California; g Department of Psychology, University of California, Los Angeles, Los Angeles, California; h Department of Psychological and Brain Sciences, Dartmouth College, Hanover, New Hampshire; i American Brain Stimulation Clinic, Del Mar, California

**Keywords:** Amygdala, Emotional reactivity, Low-intensity transcranial focused ultrasound (TFUS), Noninvasive deep brain stimulation, Psychophysiology

## Abstract

**Background:**

The amygdala is highly implicated in an array of psychiatric disorders but is not accessible using currently available noninvasive neuromodulatory techniques. Low-intensity transcranial focused ultrasound (TFUS) is a neuromodulatory technique that has the capability of reaching subcortical regions noninvasively.

**Methods:**

We studied healthy older adult participants (*N* = 21, ages 48–79 years) who received TFUS targeting the right amygdala and left entorhinal cortex (active control region) using a 2-visit within-participant crossover design. Before and after TFUS, behavioral measures were collected via the State-Trait Anxiety Inventory and an emotional reactivity and regulation task utilizing neutral and negatively valenced images from the International Affective Picture System. Heart rate and self-reported emotional valence and arousal were measured during the emotional reactivity and regulation task to investigate subjective and physiological responses to the task.

**Results:**

Significant increases in both self-reported arousal in response to negative images and heart rate during emotional reactivity and regulation task intertrial intervals were observed when TFUS targeted the amygdala; these changes were not evident when the entorhinal cortex was targeted. No significant changes were found for state anxiety, self-reported valence to the negative images, cardiac response to the negative images, or emotion regulation.

**Conclusions:**

The results of this study provide preliminary evidence that a single session of TFUS targeting the amygdala may alter psychophysiological and subjective emotional responses, indicating some potential for future neuropsychiatric applications. However, more work on TFUS parameters and targeting optimization is necessary to determine how to elicit changes in a more clinically advantageous way.

## Plain Language Summary

Transcranial focused ultrasound (TFUS) is an emerging brain stimulation technique with the ability to noninvasively alter the activity of deep brain regions. Studying the potential for TFUS to alter behavioral response and processing, this study employed MRI-guided TFUS targeting the right amygdala in older adults. We found that TFUS targeting the right amygdala increased self-reported arousal in response to negative images, providing preliminary evidence that a single session of TFUS may be capable of affecting emotional reactivity.

Anxiety disorders affect more than 45 million people worldwide (1), with numbers increasing in the face of the COVID-19 pandemic (2); however, many individuals fail to respond to existing first-line treatments (3,4). Anxiety disorders substantially impair functioning and quality of life (5,6). They have also been found to precede the onset of depressive disorders (7) and substance use disorders (8) and markedly increase their likelihood of occurrence (9,10). Uncontrolled anxiety disorders in older adults have also been associated with accelerated cognitive decline (11). Nearly 40% of patients do not respond to first-line treatments [e.g., cognitive behavioral therapy and pharmacological interventions (12)], highlighting the necessity to advance alternative and augmentative treatments. Given the increasing rates of anxiety disorders and the dangers and comorbidities that they are associated with, developing accessible, safe, and efficacious treatments for anxiety disorders is more pertinent than ever.

Advancements in neuromodulation show promising potential to selectively modulate neural activity, extending beyond the bounds of current psychiatric and neurologic treatments. Neuromodulation methods such as repetitive transcranial magnetic stimulation, transcranial direct current stimulation, and electroconvulsive therapy have been investigated as potential treatments for anxiety disorders and have shown some clinical promise, particularly for generalized anxiety disorder (13). However, these techniques are limited by their inability to reach the subcortical brain regions that are more directly implicated in these disorders, instead targeting functionally associated regions in the cortex or providing diffuse stimulation to the brain as a whole (14, 15, 16). Such subcortical regions include the amygdala, which has been shown to be dysregulated in people with anxiety disorders (17).

Amygdala circuits are known to be central to a wide range of processes including anxiety, fear processing, and emotional regulation (17). An array of studies has provided evidence for the amygdala’s role in psychiatric disorders, including posttraumatic stress disorder, obsessive-compulsive disorder, generalized anxiety disorder, major depressive disorder, and substance use disorder, as well as in emotional processing and reactivity (17, 18, 19, 20, 21, 22). Human and animal studies have also identified the association of the amygdala with the acquisition, storage, and expression of conditioned fear learning (23). Therefore, the amygdala is an excellent candidate for studying subcortical targeting and the impacts of transcranial focused ultrasound (TFUS) interventions.

Low-intensity TFUS is a novel noninvasive brain stimulation method with preliminary promise for targeting deep brain structures with high spatial resolution (24,25). Low-intensity generally refers to the magnitude of intensity at or below that of diagnostic ultrasound (26) and within the current Food and Drug Administration safety guidelines. This is in contrast to high-intensity focused ultrasound, which refers to intensities that are known to cause permanent tissue damage, predominantly through heating and/or cavitation methods (27). Low-intensity TFUS has shown the potential to modulate subcortical regions, such as the amygdala, with a high degree of specificity compared to other noninvasive brain stimulation techniques while still maintaining the potential to demonstrate a comparably high safety profile (28, 29, 30, 31). As such, TFUS offers a unique and exciting potential to physically expand the capacities of noninvasive brain stimulation therapies for neuropsychiatric disorders.

The first of its kind, this study explored the potential physiological and subjective emotional impacts of noninvasive deep brain neuromodulation of the amygdala with TFUS in healthy older adults. In this study, TFUS was also administered to the entorhinal cortex, both to create an active control condition to compare to the amygdala condition and to investigate the impact of entorhinal cortex TFUS on learning and memory. This article focuses on the impacts of TFUS on anxiety and emotional reactivity. The effects of this intervention on perfusion and functional connectivity were previously reported for the same subject sample in Kuhn *et al.* (32).

## Methods and Materials

### Ethics

All participants from the Lifespan Human Connectome Project for Typically Aging Adults (HCPA) study had consented to be contacted for potential participation in other research studies at the University of California, Los Angeles (UCLA). Before participant enrollment and data collection, this study was registered at ClinicalTrials.gov (NCT03717922) and approved by the UCLA Institutional Review Board (IRB #18-000978). All participants provided written informed consent to participate in the study. All research procedures were performed in accordance with the Declaration of Helsinki.

### Criteria

Following HCPA screening criteria [see Section 2.6 of Bookheimer *et al.* (33)], extensive screenings were administered over the phone to exclude individuals with medical or psychiatric disorders such as any history of brain injury or stroke, current use of psychotropic medication, and depression requiring treatment for 12 months or longer during the past 5 years. The screening process did not explicitly include screening for psychopathology. See Table 1, additional screening measures are detailed in the Supplement.

**Table 1:** Demographic Characteristics of the Study Sample (*N* = 21)

Characteristic	*n* (%), Mean ± SD, or *n*
Race	
Asian	2 (9.5%)
Black	3 (14.3%)
Latinx	8 (38.1%)
White	8 (38.1%)
Gender, Female/Male	
Female	11 (52.4%)
Male	10 (47.6%)
Age, Years	
Female	58.45 ± 5.03
Male	63.10 ± 10.64
Total	60.67 ± 8.52
Exclusions Post-Enrollment	4

Four of the 21 enrolled participants were excluded due to the inability to complete their second visit: 3 due to an unexpected scan center closure and 1 due to the COVID-19 pandemic.

### Study Design

**Figure 1:**
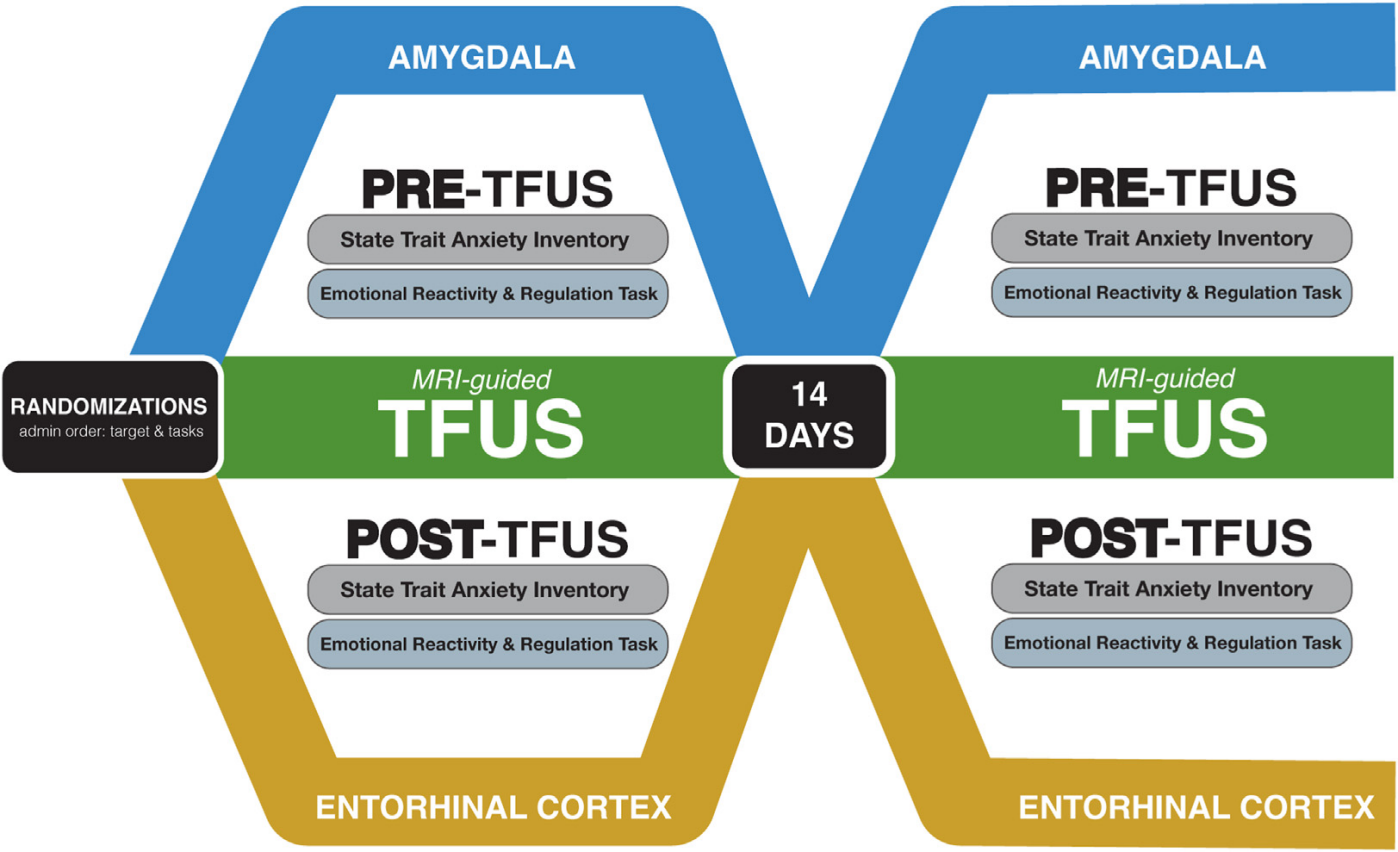
Study design. A visual representation of the randomized, double-blinded, within-participant crossover study design. Participants completed 2 study visits separated by 14 days targeting either the right amygdala or left entorhinal cortex. Examples of amygdala and entorhinal cortex transcranial focused ultrasound (TFUS) targeting are provided in Figure 2, and a chart detailing sample demographics is provided in Table 1. MRI, magnetic resonance imaging.

Utilizing a double-blinded cross-over design, each participant underwent 1 visit targeting the right amygdala and 1 visit targeting the entorhinal cortex. Neuroimaging data was collected before, during, and after in-scanner TFUS, and behavioral measures were collected outside the scanner before and after TFUS. Visits were spaced 14 days apart because it was hypothesized that the effects of a single sonication are likely to have abated within 2 weeks. However, to control for potential order or carryover effects, the region that was targeted for each visit was randomized and counterbalanced across participants (Figure 1).

### TFUS Protocols

TFUS was conducted in the Siemens 3T magnetic resonance imaging (MRI) scanner at the UCLA Center for Cognitive Neuroscience utilizing the MRI-compatible BrainSonix Corp. BX Pulsar 1002 focused ultrasound 65- and 55-mm transducers.

#### TFUS Paradigms

Both TFUS paradigms used a 5% duty cycle with a fundamental frequency of 0.65 MHz and Ispta.3 of 720 mW/cm^2^. Ispta.3 is the derated spatial peak temporal average intensity and refers to the time-averaged acoustic intensity over 1 pulse repetition period after applying the derating equation with a derating factor of 0.3 dB/cm-MHz.

For TFUS targeting the amygdala, a pulse repetition frequency of 10 Hz and a pulse width of 5 ms were used because these parameters were hypothesized to inhibit activity (25), and disruption of amygdala activity is hypothesized to be beneficial in treating anxiety disorders (34). The right amygdala was targeted based on literature suggesting that it is more heavily involved in the processing of negatively valenced emotions than the left amygdala (35). Conversely, TFUS of the entorhinal cortex used a pulse repetition frequency of 100 Hz and a pulse width of 0.5 ms, parameters that have been hypothesized to increase activity (36) because it has been hypothesized that excitation of the entorhinal cortex improves learning and memory, which is an additional aim of the broader project (37). The left entorhinal cortex was selected because it is believed to play a greater role in memory formation than the right entorhinal cortex (38), as well as to limit the likelihood of impacting the control region when targeting the active region and vice versa. Older adults were chosen as the population for this study to maximize the potential generalizability of the findings from the entorhinal cortex arm to populations with age-related cognitive decline. Additional background on target selection is available in the Supplemental Methods.

#### MRI-Guided TFUS Neuronavigation

**Figure 2:**
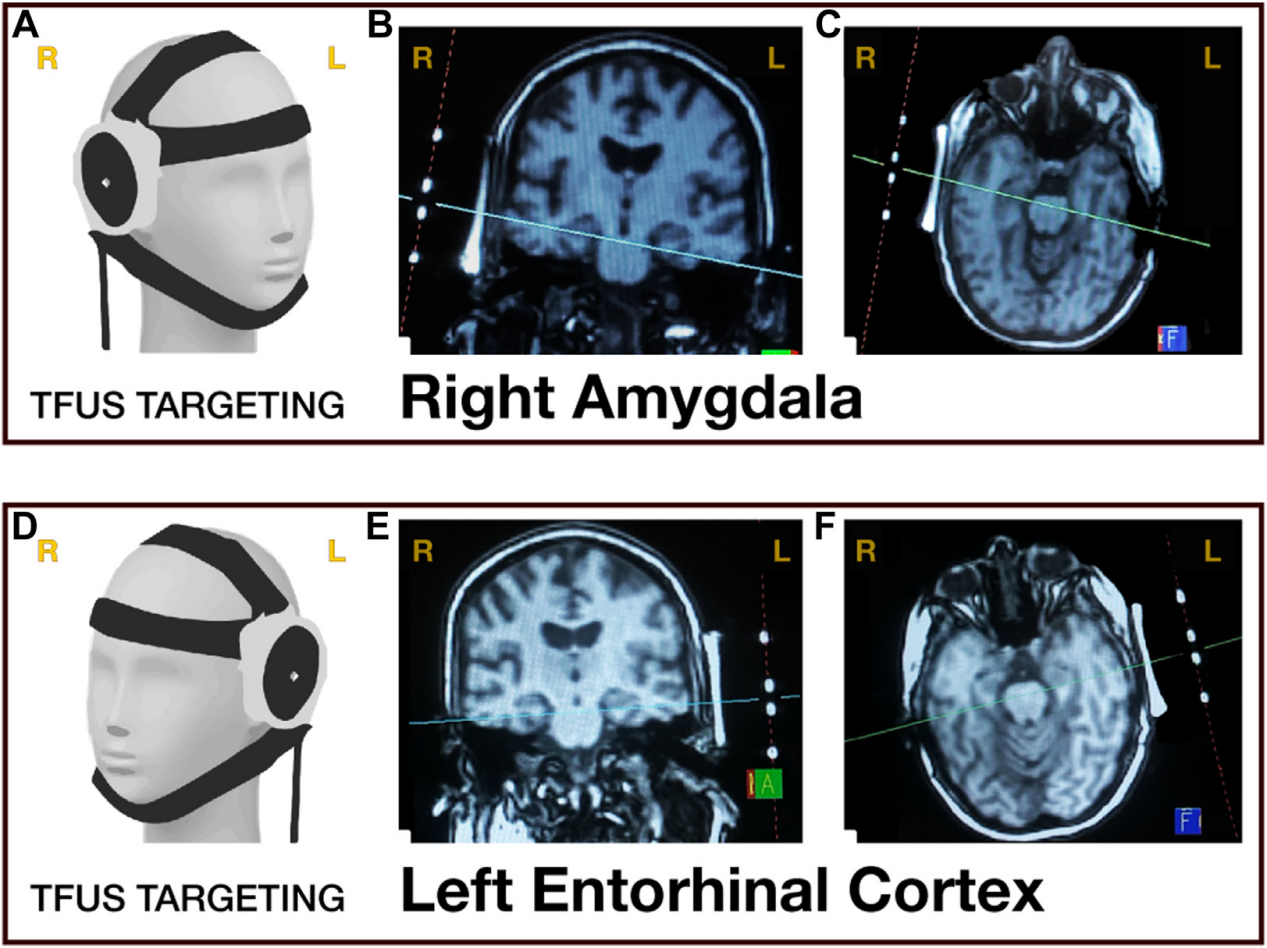
Transcranial focused ultrasound (TFUS) neuromodulation targeting. Visualization of transducer placement on a 3-dimensional model when targeting the right amygdala **(A)** and left entorhinal cortex **(D)**. Examples of magnetic resonance imaging–console-guided targeting using transducer fiducial markers are provided in the coronal view **(B, E)** and axial view **(C, F)**. Please note that the right and left are flipped (reversed) as noted in yellow letters at the top of each image. Figure adapted from Kuhn *et al.* (32). L, left; R, right.

Participants were placed in the MRI, and the transducer was secured on their head with elastic straps (Figure 2A, D). A structural localizer image was collected with a field of view that included both the participant’s head and the MRI fiducial markers built into the transducer. Siemens MRI console tools were then used to visualize the trajectory of the transducer relative to the brain based on the fiducial markers (Figure 2B, C, E, F). The transducer was then adjusted, and this process was repeated until the transducer was confirmed by the principal investigator to be accurately aimed at the target region. Localizer sequence details are further detailed in the Supplemental Methods.

### Assessments

All behavioral and psychophysiological assessments described below were collected outside of the MRI scanner in a separate testing room before and after TFUS (Figure 1).

#### State-Trait Anxiety Inventory

The single form of the State-Trait Anxiety Inventory (39) was administered to assess state and trait anxiety pre- and post-TFUS. Trait anxiety was excluded from the main analyses due to its short-term stability but is included in the Supplement.

#### Emotional Reactivity and Regulation Task

**Figure 3:**
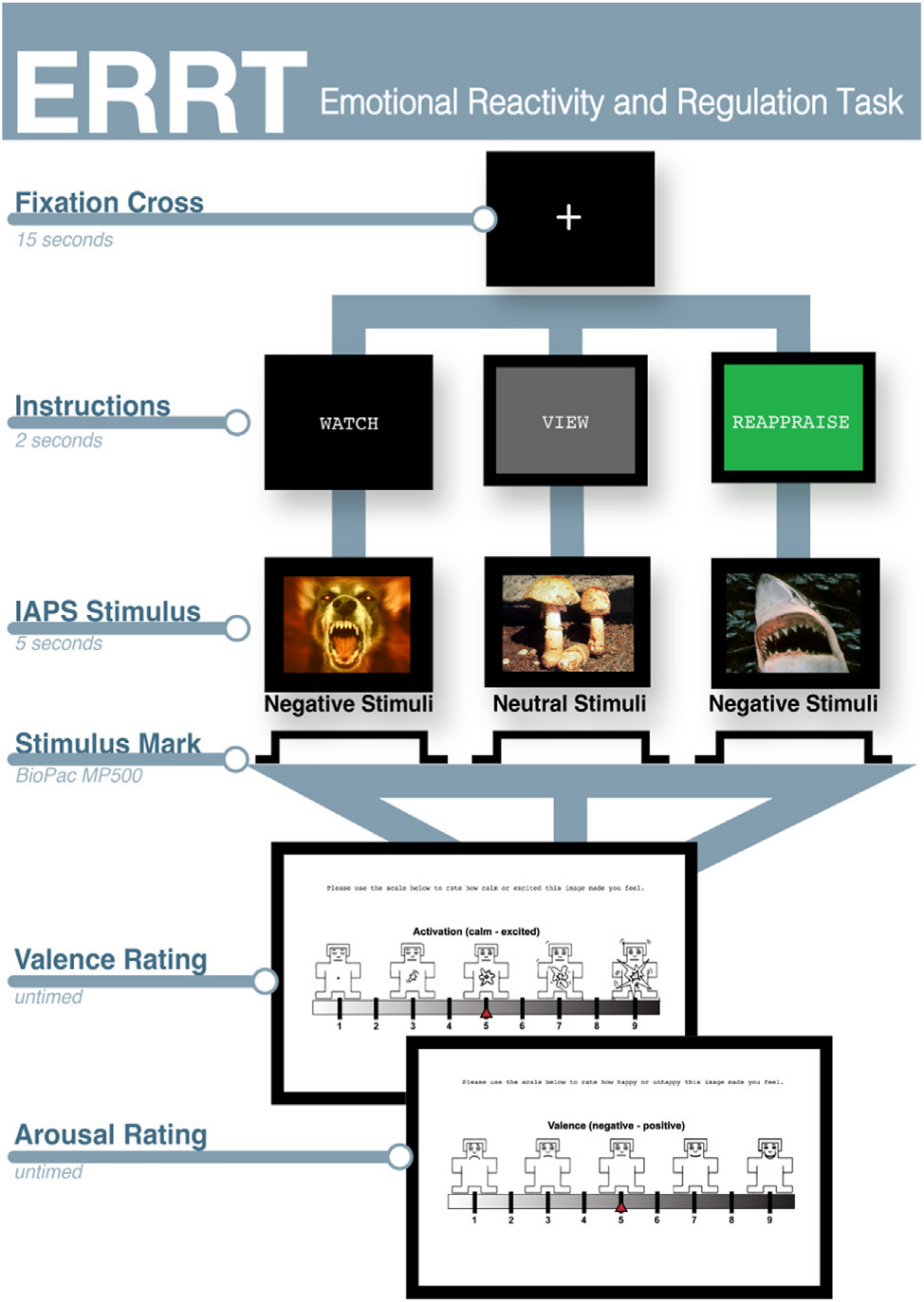
Emotional reactivity and regulation task (ERRT) design. Each ERRT trial began with a fixation cross (15 s) followed by an instruction screen (2 s) followed by an International Affective Picture System (IAPS) image occupying 80% of the screen (5 s) and ended with untimed self-ratings for valence and arousal. The stimulus and corresponding instruction type (WATCH-negative, VIEW-neutral, and REAPPRAISE-negative) varied on each trial. To assess valence and arousal, participants were asked to rate each IAPS image using the affective rating system ranging from 1 (least) to 9 (most) via the Self-Assessment Manikin created by Lang *et. al.* (56, 57, 58). Valence ratings ranged from 1 (negative) to 9 (positive), and arousal ratings ranged from 1 (calm) to 9 (exciting) (see Figure S2A, B).

The emotional reactivity and regulation task (ERRT) examined how participants responded to different types of emotionally evocative image stimuli from the International Affective Picture System (IAPS). The task included 3 types of instructions before participants viewed images: instructions about how to 1) passively view a negative image (WATCH), 2) passively view a neutral image (VIEW), or 3) actively reappraise a negative image (REAPPRAISE) (Figure 3). Four distinct ERRT forms with discrete stimuli were administered in a randomized, counterbalanced order before and after each TFUS session across both study visits. Each ERRT form contained 32 unique images: 8 neutral VIEW and 16 negative WATCH images that also appeared in the REAPPRAISE condition and 8 negative images that were unique to the REAPPRAISE condition. ERRT was created and administered using EPrime 2.0 (Psychology Software Tools). These images were repeated for a total of 96 image presentations for each administration of the ERRT.

Following each stimuli presentation, participants’ emotional responses were quantified using the Self-Assessment Manikin scale, which measures arousal (calm to excited) and valence (unhappy to happy) on a scale from 1 to 9. Emotional reactivity was assessed by comparing responses to negative WATCH and neutral VIEW images, while emotional regulation was evaluated by comparing responses to negative REAPPRAISE and negative WATCH images.

### Simultaneous ERRT Psychophysiological Data Collection

In each IAPS trial, interbeat intervals (indicating heart rate) were measured in ms using the BioPac MP150 system, BioNomadix Wireless Electrocardiogram (ECG) Amplifier, USB TTL, and AcqKnowledge version 4.2 software (BioPac Systems Inc.) for ECG collection (see the Supplemental Methods for setup). During data collection, a trigger marker was recorded in the ECG file for each stimulus presentation. After data collection, ECG data was filtered to remove noise using AcqKnowledge version 4.2 software by applying 40 Hz low-pass, 50 Hz notch, and 0.50 Hz high-pass filters. The Autonomic Nervous System Laboratory (version 2.6, University of Basel, Switzerland) was used to extract interbeat interval data. Average interbeat intervals were calculated for 5 seconds before image onset to determine resting heart rate and during image display. Cardiac change analysis involved subtracting the interbeat interval during the presentation of each IAPS image from the preceding intertrial interval. Implausible heart rates (below 30 or above 200 beats per minute) were excluded from analyses.

### Statistical Analyses

Multilevel modeling was conducted with Stata/MP version 15.1, utilizing repeated measures (level 1) within participants (level 2). Level 1 factors included TFUS target (amygdala, entorhinal cortex), pre/post (pre-TFUS, post-TFUS), and instructions (view during neutral image, view during negative image, reappraise negative images). This structure was used for analyses of self-reported arousal/valence and cardiac change. Analysis of interbeat intervals during intertrial intervals was conducted using the same model, but without the “Instructions” variable because intertrial intervals were independent of the image viewing condition. To control for familywise error, Holm–Bonferroni correction was applied within domains (indicated by results subheadings) to adjust the alpha level required to declare statistical significance.

## Results

### State-Trait Anxiety Inventory

**Figure 4:**
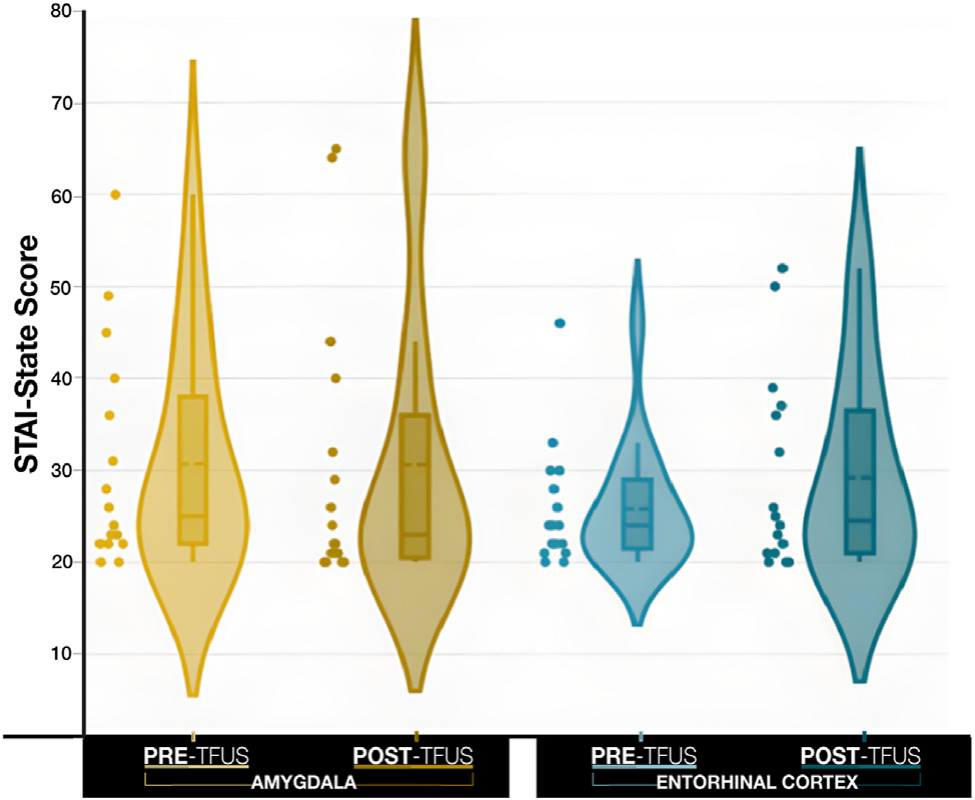
Mean State-Trait Anxiety Inventory (STAI) State score pre- and post-transcranial focused ultrasound (TFUS) targeting of the amygdala (yellow) vs. the entorhinal cortex (blue). The horizontal dashed line in the box plot indicates the mean (see Table 2). The horizontal solid line in the box plot indicates the median. No effects involving pre/post were statistically significant (*p*s > .239).

The effects of factors pre/post (pre-TFUS, post-TFUS) and TFUS target (amygdala, entorhinal cortex) on self-reported state anxiety are shown in Figure 4. No effects involving pre/post were statistically significant (*p*s > .275) (Table S2A). Descriptive statistics are reported in Table 2.

**Table 2:** STAI-State and STAI-Trait Scores Summary

	Amygdala			Entorhinal Cortex		
	Pre-TFUS	Post-TFUS	Difference	Pre-TFUS	Post-TFUS	Difference
STAI-State						
Mean	30.69	30.63	−0.06	25.81	29.25	3.44
Minimum	20.00	20.00	−20.00	20.00	20.00	−25.00
Maximum	60.00	65.00	28.00	46.00	52.00	28.00
SD	±12.00	±15.09	±11.79	±6.67	±10.64	±12.14
STAI-Trait						
Mean	35.44	33.88	−1.56	33.56	33.60	0.20
Minimum	22.00	20.00	−20.00	22.00	20.00	−3.00
Maximum	50.00	56.00	7.00	50.00	54.00	6.00
SD	±10.71	±11.41	±6.41	±9.99	±11.84	±2.68

Descriptive statistics for STAI-State and STAI-Trait assessments pre-TFUS and post-TFUS. Baseline (pre) and post-TFUS data by individual participants is available in Table S3A and S3B. Higher STAI scores indicate higher levels of anxiety; STAI scores range between 20 and 80. Individuals with STAI scores of 20–37 are classified as no to low anxiety; those with scores of 38–44 are classified as moderate anxiety; and those with scores of 44–80 are classified as high anxiety (40).

STAI, State-Trait Anxiety Inventory; TFUS, transcranial focused ultrasound.

### Emotional Reactivity Task

During the ERRT, multiple responses to the IAPS images were measured, including self-reported arousal, self-reported valence, and cardiac response measured by the average interbeat interval of the 5-second poststimulus (in 1-s intervals) subtracted by the average interbeat interval of the last 5 seconds of the preceding intertrial interval. There were no interactions involving the 1-second interbeat intervals during IAPS images, so interbeat intervals were averaged across all 5 seconds separately for each stimulus.

**Figure 5:**
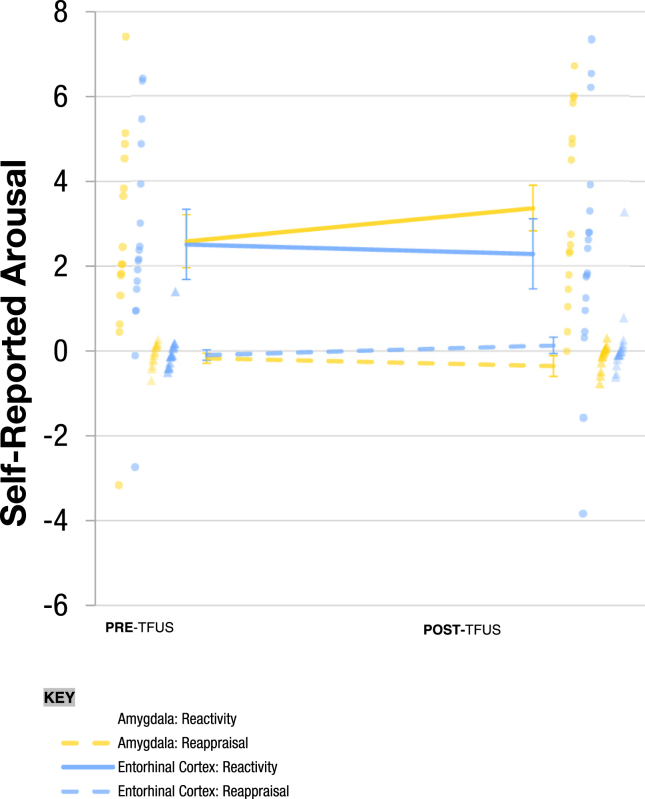
Self-reported arousal rating in response to emotional reactivity and regulation task stimuli pre- and post-transcranial focused ultrasound (TFUS) targeting of the amygdala (yellow) vs. the entorhinal cortex (blue). Reactivity refers to the contrast between viewing neutral images and viewing negative images. Reappraisal refers to the contrast between viewing negative images and reappraising negative images. Individual dots represent individual observations for each participant and each condition (e.g., amygdala: reactivity); circles represent reactivity observations, and triangles represent reappraisal observations. Individual scores are available in Table S3A, and individual changes are plotted in Figure S5A.

For self-reported arousal, Figure 5 shows the effects of factors pre/post (pre-TFUS, post-TFUS), TFUS target (amygdala, entorhinal cortex), and instructions (view neutral images, view negative images, reappraise negative images) to IAPS images. There was a statistically significant pre/post × target × instructions interaction (χ^2^_2_ = 15.99, *p* < .001, *f* = 0.051). Simple effects revealed an increase in self-reported arousal during the negative emotional reactivity contrast (viewing negative images contrasted with viewing neutral images) from pre- to post-TFUS targeting the amygdala (*Z* = 4.21, *p* < .001). Furthermore, there was a significantly greater increase in negative emotional reactivity from pre- to post-TFUS targeting the amygdala compared to pre- to post-TFUS targeting the entorhinal cortex (*Z* = −3.96, *p* < .001). All other simple effects involving pre/post were not statistically significant (*p*s > .062). Individual values for self-reported arousal are reported in Table S3A and Figure S5A.

**Figure 6:**
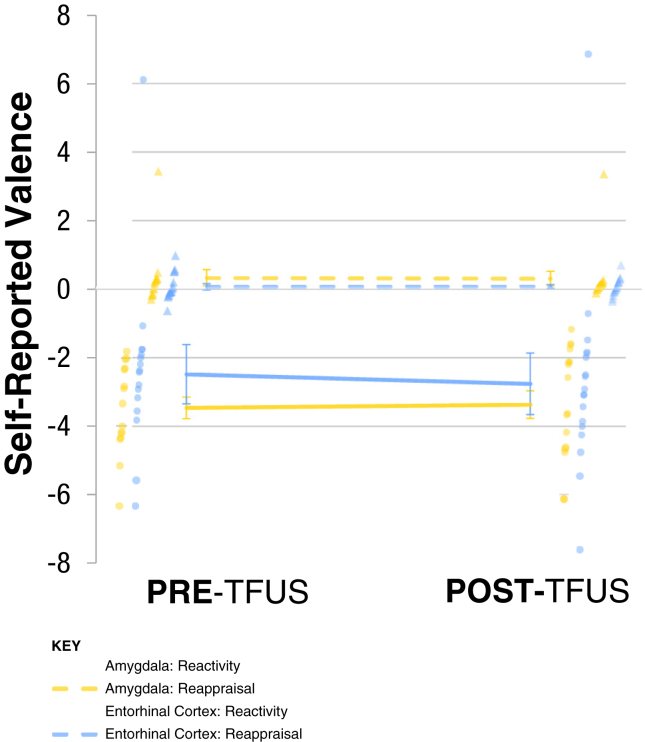
Self-reported valence in response to emotional reactivity and regulation task stimuli. Reactivity refers to the contrast between viewing neutral images and negative images pre- and post-transcranial focused ultrasound (TFUS) targeting of the amygdala (yellow) vs. the entorhinal cortex (blue). Individual values are depicted as translucent dots. Valence was reported following 5 seconds of stimulus presentation using an ordinal scale ranging from 1 to 9, where 1 is unhappy and 9 is happy (see Figure S2A). Effects of factors pre/post (pre-TFUS, post-TFUS), TFUS target (amygdala, entorhinal cortex), and instructions (“VIEW” neutral images, “WATCH” negative images, “REAPPRAISE” negative images) on the self-reported valence of International Affective Picture System images. No effects including the factor pre/post were statistically significant (*p*s > .162). Thus, there were no effects on negative emotional reactivity or negative emotional regulation. Reappraisal refers to the contrast between viewing negative images and reappraising negative images. Complete data is available in Table S3B.

For self-reported valence, Figure 6 shows the effects of factors pre/post (pre-TFUS, post-TFUS), TFUS target (amygdala, entorhinal cortex), and instructions (viewing neutral, viewing negative, reappraising negative images) on the self-reported valence of IAPS images. No effects including the factor pre/post were statistically significant (*p*s > .162). Thus, there were no effects on negative emotional reactivity or negative emotional regulation. Individual values for self-reported valence are reported in Table S3B.

For cardiac change in response to IAPS images, we found a significant pre/post (pre-TFUS, post-TFUS) × TFUS target (amygdala, entorhinal cortex) interaction (χ^2^_1_ = 5.42, *p* = .019, *f* = 0.174). However, it should be noted that there were significant differences between targets at pre-TFUS and no differences post-TFUS, reflecting a convergence of entorhinal cortex and amygdala cardiac change after TFUS. There were no significant effects involving “instructions” (viewing neutral images, viewing negative images, reappraising negative images). See the Supplement (Figure S3A, B) for details.

### Cardiac Activity During Intertrial Intervals

**Figure 7:**
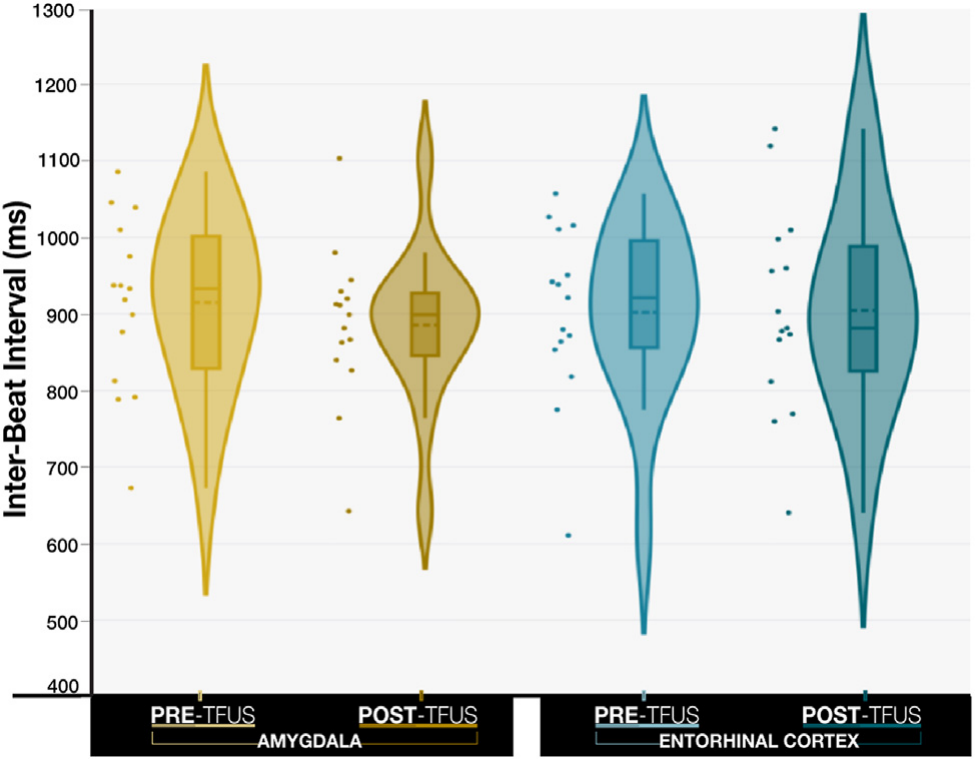
Aggregated cardiac activity at rest (during International Affective Picture System intertrial intervals) pre- and post-transcranial focused ultrasound (TFUS) targeting of the amygdala (yellow) vs. the entorhinal cortex (blue). Effects involving TFUS target (amygdala, entorhinal cortex) and pre/post (pre-TFUS, post-TFUS) on the average interbeat interval between International Affective Picture System trials. TFUS targeting the amygdala decreased interbeat intervals (i.e., increased heart rate) during intertrial intervals of the mildly aversive International Affective Picture System task. Increased intertrial intervals indicate a lower heart rate. The horizontal dashed line in the box plot indicates the mean. The horizontal solid line in the box plot indicates the median. Individual changes are plotted in Figure S4A.

Cardiac activity was measured during the intertrial intervals as a measure of physiological response to being in a mildly aversive context (i.e., the ERRT). Figure 7 shows the effects involving TFUS target (amygdala, entorhinal cortex) and pre/post (pre-TFUS, post-TFUS) on the average interbeat interval between IAPS trials. There was a significant interaction effect between pre/post and target (χ^2^_1_ = 19.17, *p* < .001, *f* = 0.012), which is illustrated in Figure 7. Evaluation of simple effects revealed a baseline (pre-TFUS) difference in the intertrial interval interbeat interval between the amygdala and entorhinal cortex (*Z* = −7.39, *p* < .001), such that the intertrial interval interbeat interval was higher before amygdala sonication than before entorhinal cortex sonication. Additionally, there was a significant decrease in intertrial interval interbeat interval (*Z* = −10.69, *p* < .001) pre- to post-TFUS targeting the amygdala, whereas TFUS targeting the entorhinal cortex sonication demonstrated no pre- to post-TFUS change (*Z* = 0.79, *p* = .429). Finally, intertrial interval interbeat interval was significantly lower post-amygdala sonication than post-entorhinal cortex sonication (*Z* = 3.59, *p* < .001). In sum, sonication of the amygdala decreased interbeat intervals (i.e., increased heart rate) during intertrial intervals of the mildly aversive IAPS task.

### Adverse Events

No serious adverse events were reported. One participant experienced a brief period of irritability and tearfulness shortly after TFUS targeting the amygdala; this had mostly resolved by the end of the session and fully resolved within 3 days of the amygdala TFUS session. It is unclear whether this was related to the TFUS or to unrelated external factors. No other adverse events were reported or evidenced by this or any other participant.

## Discussion

This study investigated the effects of TFUS targeting the right amygdala on state anxiety, emotional reactivity, and emotional regulation (cognitive reappraisal) using active TFUS targeting the left entorhinal cortex as a control condition. While no significant changes were found for state anxiety or emotional regulation, changes were found for both self-reported arousal to negative versus neutral images and heart rate during intertrial intervals of a mildly aversive ERRT.

More specifically, participants reported a significantly greater increase in self-reported arousal when viewing negative images (compared to neutral images) from pre- to post-TFUS when the amygdala was targeted than when the entorhinal cortex was targeted. This difference was driven by an increase in self-reported arousal across amygdala sonication in contrast to minimal changes across entorhinal cortex sonication. Additionally, while no changes in heart rate were observed in response to negative versus neutral images (or reappraising vs. viewing negative images), heart rate measured during intertrial intervals of the IAPS task significantly increased from pre- to post-TFUS targeting the amygdala (but not the entorhinal cortex), indicating an increase in physiological arousal during the mildly aversive task.

Overall, this data suggests that TFUS targeting the right amygdala increased reactivity to unpleasant stimuli and an unpleasant context, but not overall state anxiety. This finding, in conjunction with our previous finding of significantly increased perfusion in the amygdala post-TFUS (32), suggests that the intervention may have primarily resulted in excitation of the target region rather than the intended result of inhibition. Our observed difference in arousal responses to stimuli without perceived changes in valence implies that participants may have had a greater reactive response to images that were considered no more negative than before; this may indicate a greater reactive emotional response in the absence of changes in cognitive classification of image content. While the finding that TFUS targeting the amygdala has the capacity to change emotional reactivity demonstrates the ability to modify behavioral states with TFUS, the clinical implications of an ability to increase (rather than decrease) emotional reactivity are quite limited. This is perhaps the biggest limitation of these results and a primary direction for future work. Interpretation of these findings is further complicated by the opposite directionality of recent findings by Chou *et al.*, who used similar parameters to target the left amygdala and found a generally inhibitory effect (41). This discrepancy could be due to a variety of factors, including the lateralization of the amygdala, differences between the 2 studies in the subregions that were targeted, or methodological issues.

Determination of optimal parameters for excitation and inhibition is a critical area of ongoing research in the field due to both the novelty of the technology and the size of the parameter space. TFUS is characterized by a multitude of parameters, including intensity, pulse width, pulse repetition frequency, duty cycle, and fundamental frequency; all of these parameters have been found to impact the effects of sonication (42,43). Furthermore, recent literature suggests that neuronal responses to focused ultrasound may be cell-type-specific such that different regions of the brain may respond differently to identical parameters (44). For example, focused ultrasound pulsation in mice at 900 Hz pulse repetition frequency and 20% duty cycle was found to increase the activity of hippocampal inhibitory neurons; however, sonication of excitatory neurons in the region with identical parameters had the opposite effect, causing a decrease in the activity of these neurons (45). Because this is a very newly developing area of research, more research is needed to determine which ultrasound parameters will result in the activation or inhibition of discrete subregions within the amygdala, how the lateralization of the amygdala impacts the response to TFUS, and which amygdalar subregions are most advantageous to target in order to induce clinically beneficial changes in anxiety, emotion regulation, and/or emotional reactivity.

An important element of determining responses to TFUS at the subregion level will be to further optimize the procedures that are used for targeting. The targeting procedure for this study did not account for participant-specific variation in skull structure, which can cause the TFUS beam to shift to varying degrees (46). Although methods to account for this variation are currently being developed, they are not yet computationally optimized for real-time implementation in-scanner (46). Additional work on skull refraction modeling is needed in order to enable real-time adjustment for participant-specific skull characteristics and resulting refraction. Additionally, this study targeted the broader amygdala rather than a particular subregion. As a result, it is likely that TFUS affected multiple subregions across the subject sample, including ones that could have opposing functional effects such as the centromedial and centrolateral amygdala (47). Additionally, there is a possibility that some degree of hippocampal modulation may have occurred in some participants and impacted these findings because the hippocampus is both directly adjacent to the amygdala and highly implicated in emotional reactivity (48,49).

Notably, this study found changes in subjective emotional reactivity as well as physiological arousal changes, but no changes in generalized state anxiety. This is reminiscent of some previous literature on amygdala deep brain stimulation, which similarly found changes in specific domains such as hypervigilance in the absence of generalized anxiety reductions (50). Furthermore, while both physiological and subjective emotional changes have been observed in response to brief deep brain stimulation of the amygdala in implanted electrode studies, physiological changes appear to be more reliably induced (51). However, literature on this topic is currently limited; more research is needed to determine the impact of stimulating specific subregions of the amygdala and the duration of stimulation that is necessary to more reliably induce behavioral changes.

The implications of these findings are also limited by the relatively small effect sizes despite statistical significance. This may be due in part to the fact that this was an early proof-of-concept study that therefore only administered a single session of TFUS targeting each region. The limited effect sizes associated with this study are consistent with the results of previous studies of noninvasive brain stimulation and emotional reactivity; a 2020 review of 40 sham-controlled single-session repetitive transcranial magnetic stimulation and transcranial direct current stimulation studies concluded that while providing preliminary evidence for the capacity to impact emotional reactivity, a single session of noninvasive brain stimulation was insufficient to induce reliable, clinically significant effects (52). Similarly, a recently published study on TFUS to the left amygdala found changes in brain activation during a fear task, but no significant changes in self-reported anxiety levels during that task (41). Once sonication parameters have been tuned to elicit changes in a clinically beneficial direction, a critical next step will be to move toward a multisession paradigm resembling those that have more reliably demonstrated clinically significant changes in other noninvasive brain stimulation domains (53).

Another limitation of our findings lies in the small size of the sample and the demographic characteristics of the sample that we recruited. The small sample size significantly limited the statistical power for this study, and while it was sufficient to find statistically significant changes, there may have been other effects that this sample was not sufficiently powered to detect. Furthermore, the findings in this sample of older adults with an average age in the early 60s may fail to generalize to younger populations due to aging-related changes in amygdalar structure and connectivity (54). Future research on this topic would benefit from a larger sample size and the inclusion of participants with a broader age range. Additionally, while the ideal future application of this technology would be in neurological and psychiatric conditions such as anxiety disorders, this data were collected in healthy adults, and the lack of notable anxiety and emotional dysregulation in this population at baseline may have impacted the magnitude and directionality of the changes that were observed. Given that there are a number of well-documented differences in amygdalar structure and connectivity in individuals with psychiatric conditions, it is possible that these results may not generalize outside of a healthy control sample (55). Additional studies in psychiatric populations are needed to determine whether TFUS has the capacity to alter behavior in psychiatric conditions; however, the preliminary results of the current study suggest that more research is needed to optimize parameters for more favorable behavioral changes prior to expanding into these populations.

### Conclusions

In sum, these results provide preliminary evidence that TFUS targeting the amygdala may be capable of modulating psychophysiological and subjective emotional responses during an emotional reactivity and reappraisal task in healthy older adults, indicating merit for further investigation of the technology. However, given that the TFUS protocol that was used in this study was found to increase emotional reactivity rather than decrease it, more work on optimizing sonication parameters and targeting methodology is necessary to determine effective protocols for potential clinical benefit.

## Supplementary Material

Key Resources Table
