# Supplementary material for: Transcranial Focused Ultrasound Targeting the Amygdala May Increase Psychophysiological and Subjective Negative Emotional Reactivity in Healthy Older Adults
Source: Biol Psychiatry Glob Open Sci. 2024 Jun 5;4(5):100342. doi: 10.1016/j.bpsgos.2024.100342 (PMC11293512; doi:10.1016/j.bpsgos.2024.100342)
Supplement: Supplemental Methods [file mmc2.pdf]

SUPPLEMENTARY INFORMATION

Transcranial Focused Ultrasound Targeting the Amygdala May Increase Psychophysiological and Subjective Negative Emotional Reactivity in Healthy Older Adults

Hoang-Dang *et al.*

Supplemental Materials

5. Supplemental Methods

5.1. Criteria

Participants prescribed beta-blockers were included contingent on skipping their medication for their study visits. These screenings also included an MRI eligibility screening to ensure that participants were safe to be MRI scanned. Following written consent at the first visit, the MoCA was administered to ensure cognitive eligibility with a cut-off score of 19 per HCPA protocol (1,2).

5.2. TFUS

5.2.1. TFUS Administration

The brain region targeted during each study session was randomized and counterbalanced across participants such that 43% received amygdala TFUS during the first study session and 57% received entorhinal cortex TFUS during their first session. Examples of amygdala and entorhinal cortex TFUS targeting are provided in Figure 2, as well as a chart detailing sample demographics in Table 1.

5.2.2. TFUS Target Selection

The right amygdala was chosen as the target for this study as it is believed to be involved in the processing of negatively valenced emotions to a greater degree than the left amygdala (3). This made it the most logical target for this project, given the goal of modulating anxiety and emotional reactivity to negative stimuli.

The entorhinal cortex was chosen as an active control region as it allowed the study to investigate the regional specificity of TFUS targeting and brain region engagement due to its close proximity to the amygdala. The left entorhinal cortex was chosen in particular in order to limit the likelihood of impacting the active region when trying to target the control region. Furthermore, a separate aim of this project (not reported in this manuscript) was to gauge the ability of TFUS to the left entorhinal cortex to impact learning and memory, and the left entorhinal cortex is thought to play a disproportionate role in memory formation (4). The inclusion of the left entorhinal cortex in this manner thereby enabled the investigation of both the impact of amygdala TFUS on anxiety and emotional reactivity with the entorhinal cortex as an active control, and the investigation of the impact of entorhinal cortex TFUS on learning and memory with the amygdala as an active control region.

Although the entorhinal cortex is functionally connected to the amygdala and this connection plays a role in the

emotional enhancement of memory, both in healthy and pathological (e.g. intrusive recollections in PTSD) contexts, their primary structural and functional networks are distinct. As such, these brain regions are implicated in generally distinct neurologic and psychiatric syndromes (e.g. anxiety disorders vs amnestic syndromes), and demonstrating the selective utility of tFUS targeting these two regions forms the foundation for further exploration of tFUS of these two distinct regions as a potential clinical treatment for distinct clinical phenomena.

5.2.3 Targeting Localizer Sequence

The T1-weighted localizer images used for TFUS targeting were acquired on a 3T Siemens MAGNETOM Prisma fit scanner (Siemens Medical Solution, Erlangen, Germany) with TR=3.15ms, TE=1.37ms, flip angle 8 degrees, voxel size 1.6mm x 1.625mm x 1.625mm, matrix size 128 x 160 x 160, FOV 260mm.

5.3. Behavioral & Psychophysiological Measures Pre-TFUS and Post-TFUS

Before and after each TFUS session, participants completed an emotional reactivity and regulation task (ERRT) utilizing neutral and negatively valenced images from the International Affective Pictures Set (IAPS), and self-reported emotional valence and arousal were measured in response to the stimuli. Heart rate was measured during the ERRT to investigate psychophysiological responses.

5.3.1. State-Trait Anxiety Inventory

The State-Trait Anxiety Inventory (STAI) was collected before and after each study visit to determine overall anxiety levels from before to after TFUS (Fig S1). Form Y-1 of the STAI was the most updated form available upon the beginning of this study and was used both before and after TFUS in both study visits. The STAI was completed by participants using self-ratings for the questions assessing symptoms of anxiety (Fig S1) and is broken up into two components: STAI-State (STAI-S) and STAI-Trait (STAI-T), where the STAI-S assesses anxiety symptoms at the time of administration, whereas STAI-T refers to overall "general" tendencies attributed to the longer term personality trait of personality over longer periods of time.

Given that STAI-Trait scores characterizes anxiety as a stable personality trait and the pre and post-TFUS assessments measured were approximately 3 hours apart, STAI-T scores were unlikely to shift. Thus, STAI-T scores were omitted from the main report. Scores for the STAI-S and STAI-T are summed up for a general score, but the subscores for STAI-S were reported in the main text only.

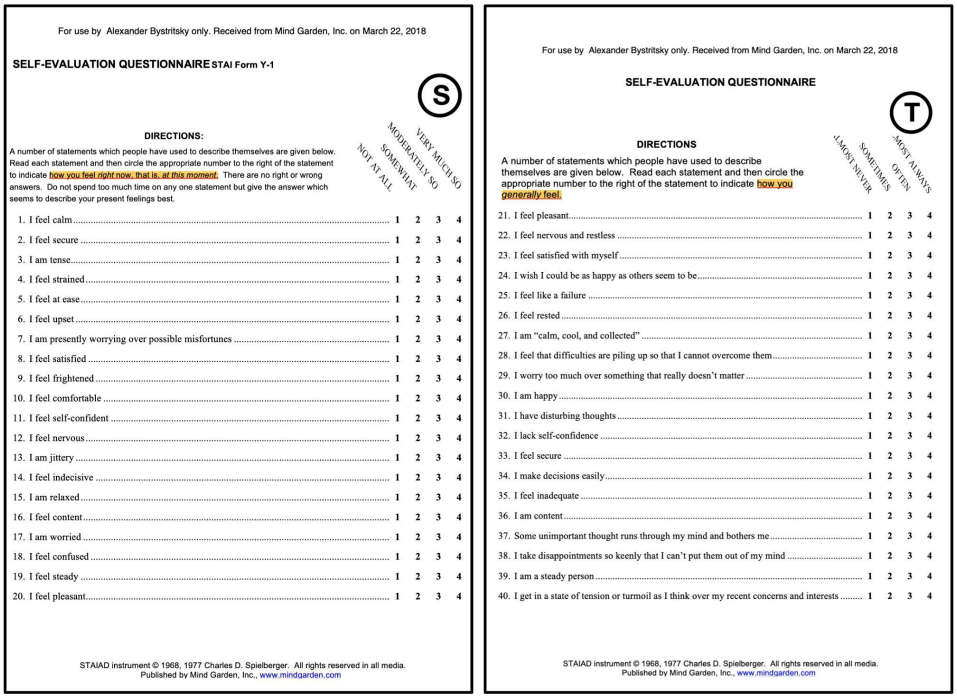

For use by Alexander Bystritsky only. Received from Mind Garden, Inc. on March 22, 2018

SELF-EVALUATION QUESTIONNAIRE STAI Form Y-1

S

DIRECTIONS:

A number of statements which people have used to describe themselves are given below. Read each statement and then circle the appropriate number to the right of the statement to indicate how you feel *right* now, that is, *at this moment*. There are no right or wrong answers. Do not spend too much time on any one statement but give the answer which seems to describe your present feelings best.

	NOT AT ALL	SOMEWHAT	MODERATELY SO	VERY MUCH SO
1. I feel calm	1	2	3	4
2. I feel secure	1	2	3	4
3. I am tense	1	2	3	4
4. I feel strained	1	2	3	4
5. I feel at ease	1	2	3	4
6. I feel upset	1	2	3	4
7. I am presently worrying over possible misfortunes	1	2	3	4
8. I feel satisfied	1	2	3	4
9. I feel frightened	1	2	3	4
10. I feel comfortable	1	2	3	4
11. I feel self-confident	1	2	3	4
12. I feel nervous	1	2	3	4
13. I am jittery	1	2	3	4
14. I feel indecisive	1	2	3	4
15. I am relaxed	1	2	3	4
16. I feel content	1	2	3	4
17. I am worried	1	2	3	4
18. I feel confused	1	2	3	4
19. I feel steady	1	2	3	4
20. I feel pleasant	1	2	3	4

STAIAD instrument © 1968, 1977 Charles D. Spielberger. All rights reserved in all media.
Published by Mind Garden, Inc., www.mindgarden.com

For use by Alexander Bystritsky only. Received from Mind Garden, Inc. on March 22, 2018

SELF-EVALUATION QUESTIONNAIRE

T

DIRECTIONS

A number of statements which people have used to describe themselves are given below. Read each statement and then circle the appropriate number to the right of the statement to indicate how you *generally* feel.

	ALMOST NEVER	SOMETIMES	OFTEN	ALMOST ALWAYS
21. I feel pleasant	1	2	3	4
22. I feel nervous and restless	1	2	3	4
23. I feel satisfied with myself	1	2	3	4
24. I wish I could be as happy as others seem to be	1	2	3	4
25. I feel like a failure	1	2	3	4
26. I feel rested	1	2	3	4
27. I am "calm, cool, and collected"	1	2	3	4
28. I feel that difficulties are piling up so that I cannot overcome them	1	2	3	4
29. I worry too much over something that really doesn't matter	1	2	3	4
30. I am happy	1	2	3	4
31. I have disturbing thoughts	1	2	3	4
32. I lack self-confidence	1	2	3	4
33. I feel secure	1	2	3	4
34. I make decisions easily	1	2	3	4
35. I feel inadequate	1	2	3	4
36. I am content	1	2	3	4
37. Some unimportant thought runs through my mind and bothers me	1	2	3	4
38. I take disappointments so keenly that I can't put them out of my mind	1	2	3	4
39. I am a steady person	1	2	3	4
40. I get in a state of tension or turmoil as I think over my recent concerns and interests	1	2	3	4

STAIAD instrument © 1968, 1977 Charles D. Spielberger. All rights reserved in all media.
Published by Mind Garden, Inc., www.mindgarden.com

Figure S1. State Trait Inventory

5.3.2. Emotional Reactivity & Regulation Task (ERRT)

5.3.2.1. ERRT Form Versioning

For the four sessions (pre/post Amygdala/Entorhinal cortex TFUS), ERRT form administration was also counterbalanced. The tasks were administered using EPrime 2.0.

5.3.2.2. ERRT Physical Set Up

Before starting the ERRT, ECG electrodes were positioned on cleaned and prepped skin of the upper right of the chest two inches below the collarbone and one inch below the bottom of the left outer ribcage on participants. ECG data was collected using the BioPac Systems Inc. BioNomadix Amplifier connected to the BioPac USB-TTL Trigger Box, AcqKnowledge v4.2 software, and EPrime 2.0. The ERRT task was administered using EPrime 2.0. Upon stimulus presentation, a trigger from the ECG via the TTL include a marker in the ECG recording in AcqKnowledge.

5.3.2.3. ERRT Administration

During stimulus onset, a mark was sent from the BioPac MP500 was recorded in the AcqKnowledge v4.2 software recording ECG data throughout the entire ERRT session.

5.3.2.4. ERRT Instructions

VIEW and WATCH trials were functionally identical in that both instructed subjects to simply attend to the image. However, we chose to use different cues for these two conditions in order to equate the four trial types in the level of anticipatory knowledge subjects had about the upcoming picture. These trials were intended to elicit unregulated forms of emotional response.

For REAPPRAISE trials, subjects were asked to feel neutral in response to the aversive image by altering their construal of the image, such as by imagining that the depicted scenario would improve over time or by adopting the perspective of a detached observer.

5.3.2.5. Stimuli

The average valence ratings for the ERRT's selected negative images per the IAPS data set by Lang et al. 2008 was 1.88 (SD ±3.69) and arousal rating average 4.16 (SD ±2.89). This task is mildly aversive due to its low valence and mid-level arousal ratings, given that these ratings are based on scales 1 (low) to 9 (high) – making these negative stimuli images relatively unpleasantly valenced (1.88 ±3.69) and mildly arousing emotionally (4.16 ±2.89). Rating averages for valence and arousal, as well as stimulus general subject matter, were maintained throughout all four task forms for both negative and neutral stimuli in ERRT forms.

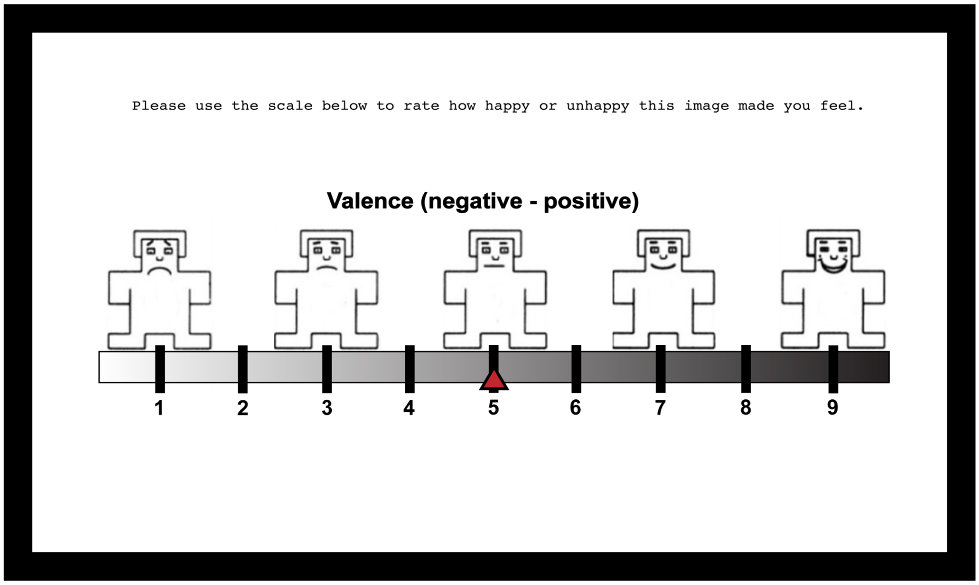

Figure S2A. Valence Rating Scale and Instruction Screen

Valence Self-Rating Screen using the Samikin Likert Rating Scale. Instructions read: "Please use the scale below to rate how happy or unhappy this image made you feel" (Lang et al. 1980, 2008). Valence rating is 1 (negative) to 9 (positive).

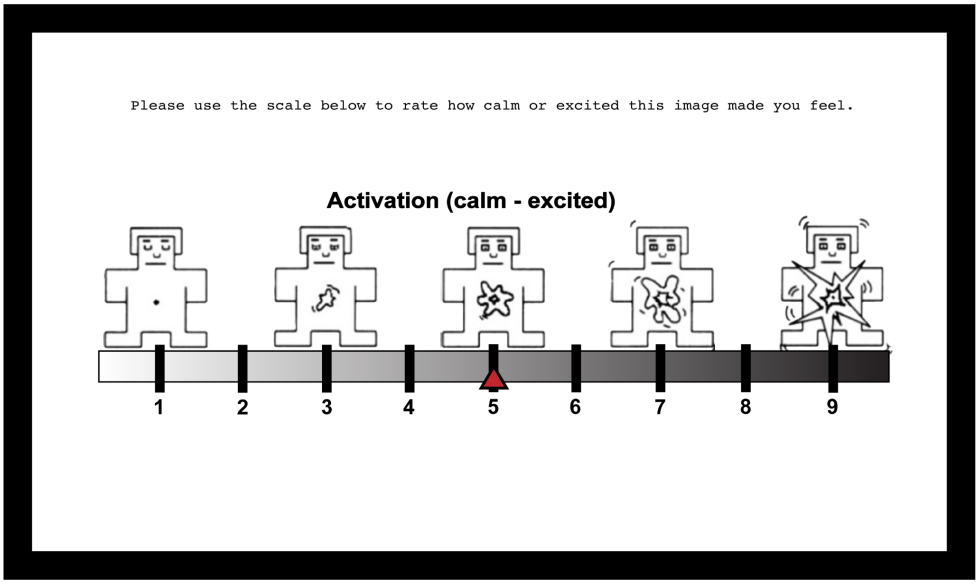

Figure S2B. Arousal Rating Scale and Instruction Screen

Arousal Self-Rating Screen using the Samikin Likert Rating Scale. Instructions read: "Please use the scale below to rate how calm or excited this image made you feel" (Lang et al. 1980, 2008). Arousal rating is 1 (calm) to 9 (excited).

6. Supplemental Results

For cardiac response to IAPS images baselined to preceding inter-trial interval, Figure S2 and Table S1 show the effects involving Pre/Post (pre-TFUS, post-TFUS), TFUS Target (amygdala, entorhinal cortex), and Instructions (view during neutral image, view during negative image, reappraise negative images) on the change in inter-beat interval from inter-trial intervals to IAPS image presentation. There was no significant effect of instructions on cardiac response to IAPS images. There was a significant interaction effect between Pre/Post and Target ($\chi^2(1) = 5.42$, $p=0.019$), indicating a significant increase in inter-beat interval pre- to post- TFUS targeting the amygdala as compared to pre- to post- TFUS targeting the entorhinal cortex. Additionally, the baselined inter-beat interval was greater post-TFUS targeting the amygdala than pre-TFUS targeting the amygdala ($Z=2.05$, $p=0.04$). Evaluation of simple effects reveals that baselined inter-beat interval pre-amygdala TFUS was significantly lower than baselined inter-beat interval pre-entorhinal cortex TFUS ($Z=3.22$, $p<0.001$), with inter-beat interval decreasing in response to stimuli pre-amygdala but increasing pre-entorhinal cortex. This difference between groups was no longer present post-TFUS. All other effects involving Pre/Post were not significant ($ps>0.133$).

Table S1A. STAI-S Total Scores.

Interaction/Main Effect	Simple Effects	df	χ^2	p	Z
Target * Pre/Post		1	1.18	0.276	
Target		1	3.37	0.066	
Pre/Post		1	0.3	0.585	

Supplementary Table S1A. STAI-S Total Scores Pre- and Post- TFUS.
No effects involving Pre/Post were statistically significant (ps>0.239)

Table S1B. Individual STAI-State Total Scores

	Amygdala			Entorhinal Cortex		
	Pre	Post	Difference	Pre	Post	Difference
	26	20	-6	21	20	-1
	49	29	-20	24	24	0
	20	20	0	46	21	-25
	28	24	-4	24	26	2
	45	65	20	28	50	22
	23	32	9	30	32	2
	22	20	-2	22	37	15
	22	21	-1	22	22	0
	40	40	0	30	36	6
	24	21	-3	20	20	0
	22	26	4	22	23	1
	20	20	0	21	20	-1
	23	22	-1	20	21	1
	36	64	28	24	52	28
	60	44	-16	26	39	13
	31	22	-9	33	25	-8
**Mean**	30.69	30.63	-0.06	25.81	29.25	3.44
**Min**	20.00	20.00	-20.00	20.00	20.00	-25.00
**Max**	60.00	65.00	28.00	46.00	52.00	28.00
**St Dev**	±12.00	±15.09	±11.79	±6.67	±10.64	±12.14

Supplementary Table S1B. STAI-S Individual Scores Pre- and Post- TFUS.
The average baseline STAI-State score was 30.69 for pre-amygdala TFUS and 25.81 for pre-entorhinal cortex TFUS with a mean change of -0.06 (SD ±11.79) post-amygdalar TFUS and +3.44 (SD ±12.14) post-entorhinal cortex TFUS.

Table S1C. Individual STAI-Trait Total Scores

	Amygdala			Entorhinal Cortex		
	Pre	Post	Difference	Pre	Post	Difference
	48	47	-1	48	50	2
	43	42	-1	34	34	0
	25	26	1	26	24	-2
	31	24	-7	23	24	1
	49	56	7	45	51	6
	46	38	-8	39	38	-1
	26	30	4	24	28	4
	22	21	-1	22	20	-2
	38	39	1	39	37	-2
	23	23	0	24	22	-2
	33	28	-5	29	26	-3
	23	20	-3	24	23	-1
	50	30	-20	50	54	4
	47	53	6	47	47	0
	24	25	1	27	26	-1
	39	40	1	36	-	-
**Mean**	35.44	33.88	-1.56	33.56	33.60	0.20
**Min**	22.00	20.00	-20.00	22.00	20.00	-3.00
**Max**	50.00	56.00	7.00	50.00	54.00	6.00
**St Dev**	±10.71	±11.41	±6.41	±9.99	±11.84	±2.68

Supplementary Table S1C. STAI-T Individual Scores Pre- and Post- TFUS.
The average baseline STAI-Trait score was 35.44 for pre-amygdala TFUS and 33.56 for pre-entorhinal cortex TFUS with a mean change of -1.56 (SD ±6.41) post-amygdala TFUS and +0.20 (SD ±2.68) post-entorhinal cortex TFUS.

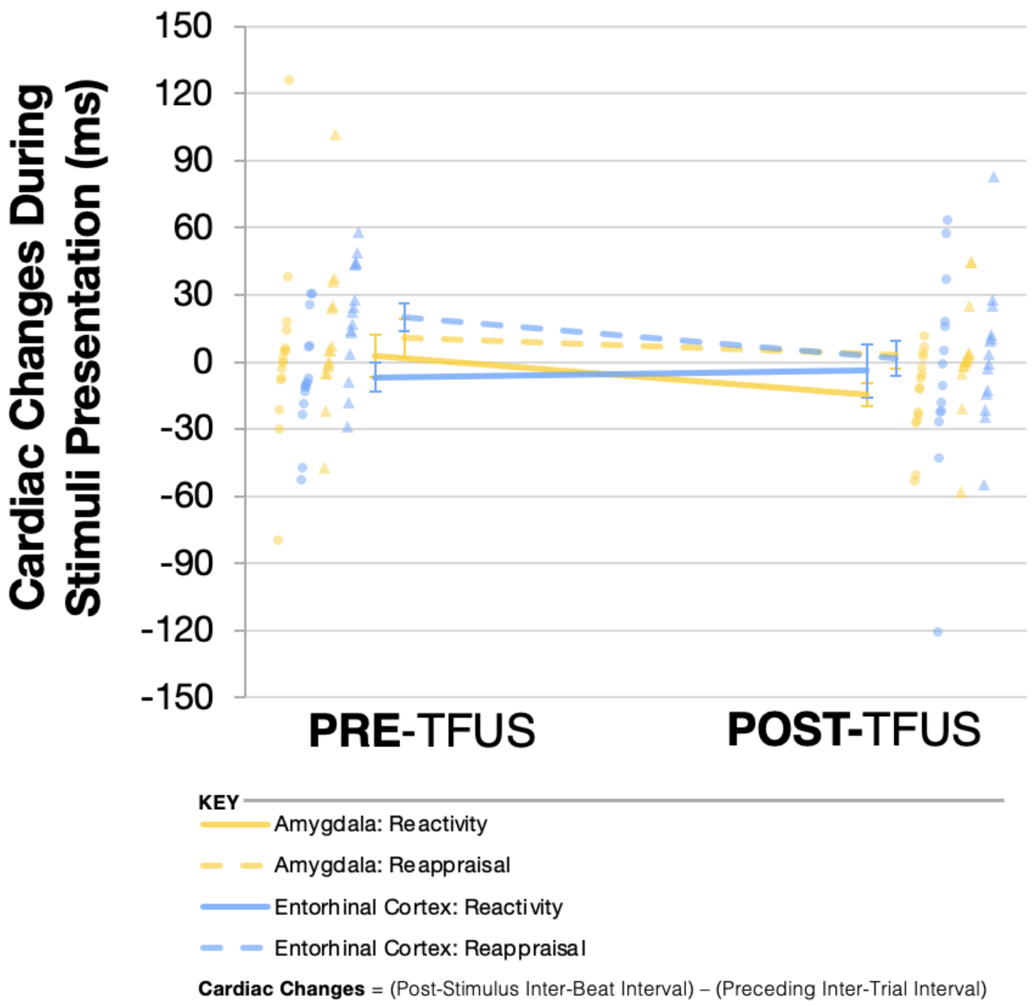

Figure S3A: Cardiac response to ERRT task before and after TFUS targeting the amygdala (yellow) vs. entorhinal cortex (blue).

"Reactivity" refers to the contrast between viewing neutral images and negative images."Reappraisal" refers to the contrast between viewing negative images and reappraising negative images. Individual dots represent individual observations for each participant and each condition (e.g., Amygdala: Reactivity); circles represent "Reactivity" observations, and triangles represent "Reappraisal" observations. For each IAPS trial, the average inter-beat interval 5-seconds post-stimulus was subtracted by the average inter-beat interval of the preceding inter-trial rest period.

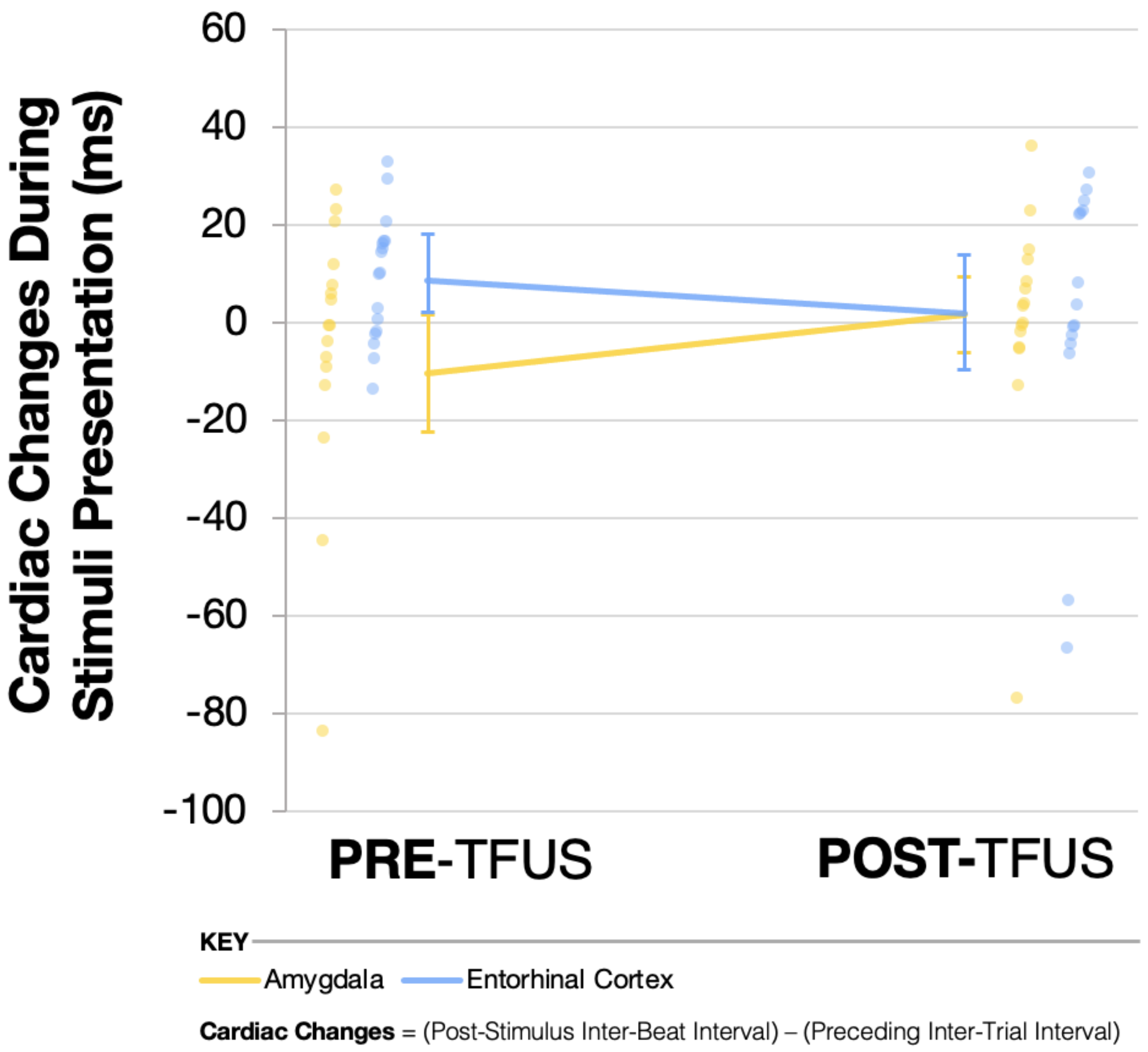

Figure S3B. Cardiac Changes During IAPS Stimuli Presentation in ERRT Task in Response TFUS Targeting Amygdala vs Entorhinal Cortex

Cardiac changes were calculated by taking the difference of the post-stimulus IBI from the preceding inter-trial intervals as specified in the Results (see Section 3). Effects are collapsed across Watch, View, and Reappraise. Individual data points represent mean cardiac change values within each participant across all IAPS images within each level of the factors. Error bars represent the standard error of the mean across all 5-second intervals for each IAPS image within each level of the factors. Note: higher inter-beat interval values indicate a decrease in heart rate.

Table S2A. Cardiac Response to IAPS Images.					
Interaction/Main Effect	Simple Effects	df	χ^2	p	Z
Target * Instructions * Pre/Post		2	0.97	0.616	
**Target * Pre/Post**		**1**	**5.42**	**0.020**	
	Entorhinal Cortex: Pre to Post			0.216	-1.24
	Amygdala: Pre to Post			**0.040**	**2.05**
	Pre: Entorhinal Cortex vs Amygdala			**< 0.001**	**3.22**
	Post: Entorhinal Cortex vs Amygdala			0.994	0.01
Instructions * Pre/Post		2	4.02	0.134	

Table S2A. Cardiac Response to IAPS Images.
"Pre/Post" indicates pre- and post-TFUS. "Target" refers to TFUS targeting the entorhinal cortex and amygdala. "Instructions" refers to viewing neutral images, viewing negative images, or reappraising negative images to be less negative. "Stimulus Timing" refers to the five 1-second intervals of time from which cardiac inter-beat intervals were extracted during the 5-second IAPS images, subtracting the 5-second inter-beat intervals prior to trial onset. Significant results are in bold. Note that only effects involving Pre/Post were included to exclusively evaluate the effect(s) of TFUS. See Supplemental Figure S4.

Table S2B. Self-Reported Arousal in Response to IAPS Stimuli.					
Interaction/Main Effect	Simple Effects	df	χ^2	p	Z
**Target * Instructions * Pre/Post**		**2**	**15.99**	**<0.001**	
	**Amygdala: (Negative-View vs Neutral-View at Pre) vs (Negative-View vs Neutral-View at Post)**			**<0.001**	**4.21**
	Entorhinal Cortex: (Negative-View vs Neutral-View at Pre) vs (Negative-View vs Neutral-View at Post)			0.191	-1.31
	Amygdala: (Negative-Reappraise vs Negative -iew at Pre) vs (Negative-Reappraise vs Negative-View at Post)			0.281	-1.08
	Entorhinal Cortex: (Negative-Reappraise vs Negative-View at Pre) vs (Negative-Reappraise vs Negative-View at Post)			0.115	1.58
	**Amygdala vs Entorhinal cortex: (Negative View vs Neutral View at Pre) vs (Negative View vs Neutral View at Post)**			**<0.001**	**-3.96**
	Amygdala vs Entorhinal Cortex: (Negative Reappraise vs Negative View at Pre) vs (Negative Reappraise vs Negative View at Post)			0.062	1.86

Table S2B. Self-reported arousal in response to IAPS stimuli.

Significant values bolded. There was a statistically significant Pre/Post x Target x Instructions interaction ($\chi^2(2) = 15.99$, $p<0.001$). Simple effects revealed an increase in negative emotional reactivity ("WATCH" negative images contrasted against "VIEW" neutral images) from pre- to post-TFUS targeting the amygdala ($Z=4.21$, $p<0.001$). Furthermore, there was a significantly greater increase in negative emotional reactivity from pre- to post-TFUS targeting the amygdala compared to pre- to post-TFUS targeting the entorhinal cortex ($Z=-3.96$, $p<0.001$). All other simple effects involving Pre/Post were not statistically significant (ps>0.062). ("WATCH", "VIEW", "REAPPRAISE") for one of three trial types: negative view (passively view a negative image; labeled "WATCH"), neutral view (passively view a neutral image; labeled "VIEW"), or negative reappraise (actively reappraise a negative image; labeled "REAPPRAISE; Figure 3).

Table S2C. Self-Reported Valence in Response to IAPS Stimuli					
Interaction/Main Effect	Simple Effects	df	χ^2	p	Z
**Target * Instructions * Pre/Post**		**2**	**3.64**	**0.162**	
Target * Pre/Post		1	0.51	**0.476**	
Instructions * Pre/Post		2	0.56	0.758	
Pre/Post		1	1.94	**0.164**	

Table S2C. Self-reported valence in response to IAPS stimuli.

No effects including the factor Pre/Post were statistically significant (ps>0.162). Thus, there were no effects on negative emotional reactivity or negative emotional regulation.

Table 2SD. Cardiac Activity Between IAPS Trials.					
Interaction/Main Effect	Simple Effects	df	χ^2	p	Z
**Target * Pre/Post**		**1**	**19.17**	**<.001**	
	**Amygdala: Pre to Post**			**<.001**	**-10.69**
	Entorhinal Cortex: Pre to Post			0.429	0.79
	**Pre: Amygdala vs Entorhinal Cortex**			**<.001**	**-7.39**
	**Post: Amygdala vs Entorhinal Cortex**			**<.001**	**3.59**

Table S2D. Cardiac activity between IAPS trials.

"Pre/Post" indicates pre- and post-TFUS. "Target" refers to TFUS targeting the entorhinal cortex and amygdala. Significant results are in bold. Evaluation of simple effects reveals a baseline (Pre-TFUS) difference in the inter-trial interval inter-beat interval between amygdala and entorhinal cortex ($Z=-7.39$, $p<0.001$), such that inter-trial interval inter-beat interval was higher before amygdala sonication than before entorhinal cortex sonication. Further, there was a significant pre-TFUS targeting post-TFUS amygdala sonication decrease in inter-trial interval inter-beat interval ($Z=-10.69$, $p<0.001$), whereas entorhinal cortex sonication demonstrated no pre- to post-TFUS change ($Z=0.79$, $p=0.429$). Finally, inter-trial interval inter-beat interval was significantly lower post-amygdala sonication than post-entorhinal cortex sonication ($Z=3.59$, $p<0.001$) (complete data available in Supplementary Table S2D). TFUS targeting the amygdala decreased inter-beat intervals (i.e., increased heart rate) during inter-trial intervals of the mildly aversive IAPS task.

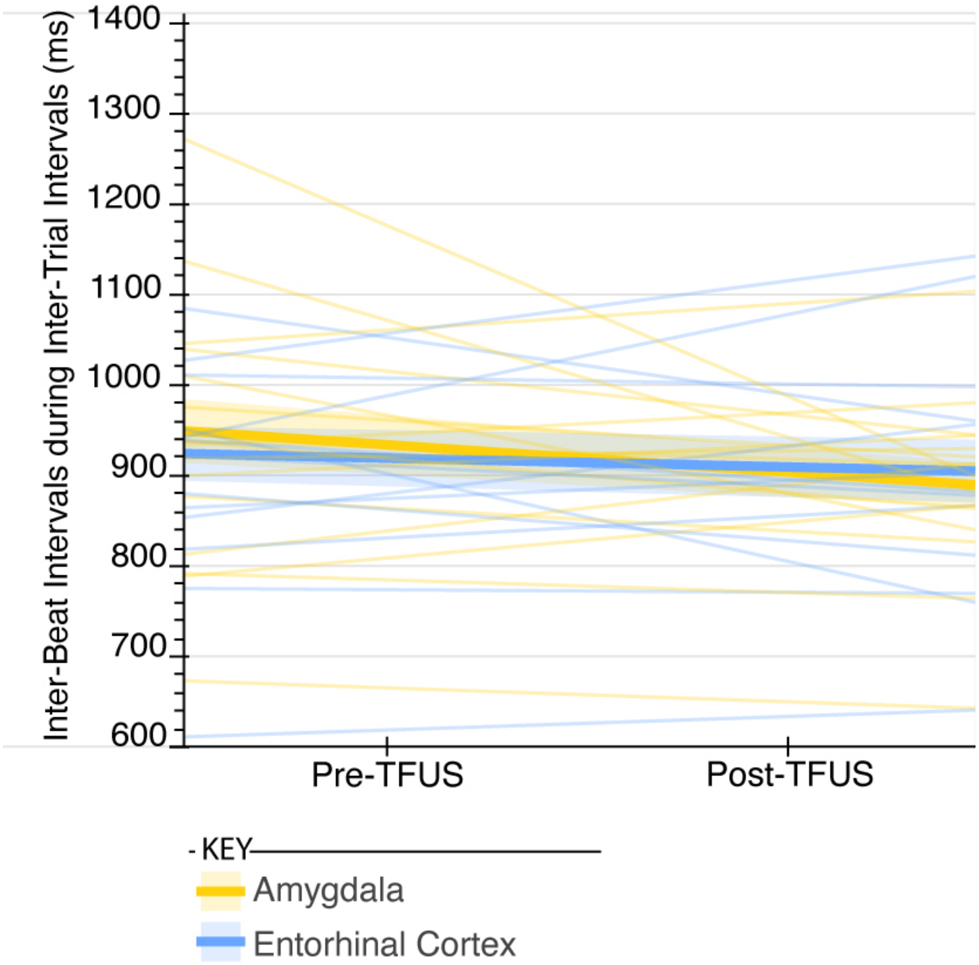

Figure S4A. Individual Participant Inter-Beat Intervals During Inter-Trial Intervals by Target

Individual inter-beat intervals during inter-trial intervals involving TFUS Target (amygdala, entorhinal cortex) and Pre/Post (pre-TFUS, post-TFUS) on the average inter-beat interval between IAPS trials. TFUS targeting the amygdala decreased inter-beat intervals (i.e., increased heart rate) during inter-trial intervals of the mildly aversive IAPS task. Increased inter-trial intervals indicate a lower heart rate. A violin plot of data is included in the main manuscript in Figure 7.

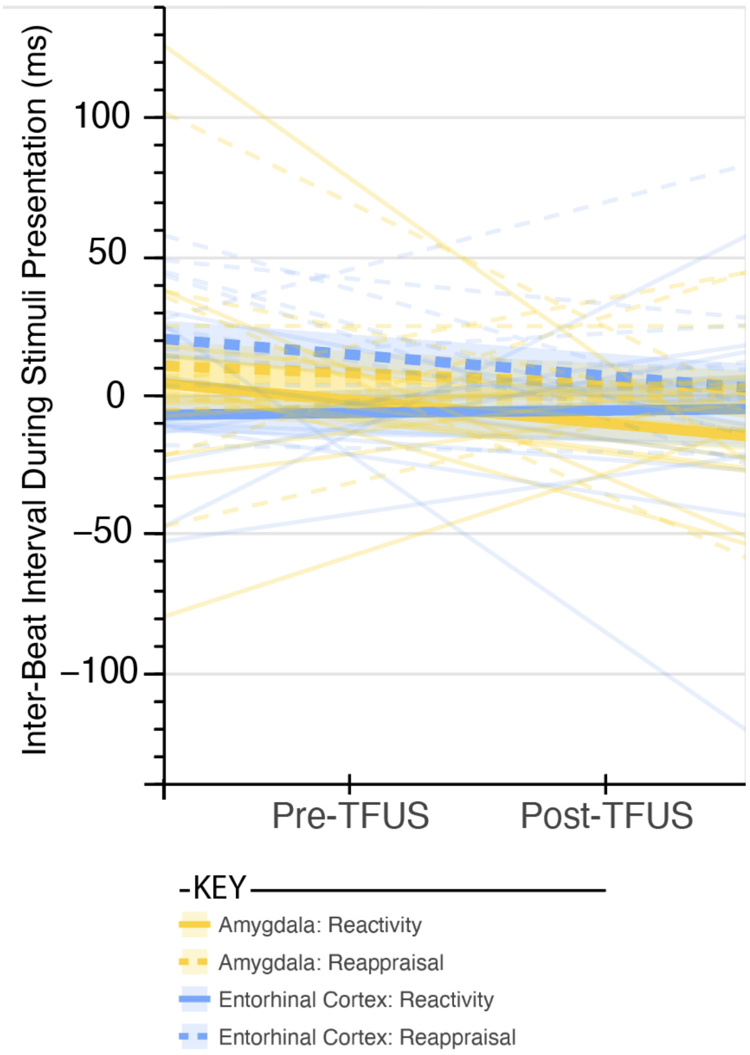

Figure S4B. Inter-Beat Interval During Stimuli Presentation

Individual inter-beat intervals during inter-trial intervals involving TFUS Target (amygdala, entorhinal cortex) and Pre/Post (pre-TFUS, post-TFUS) on the average inter-beat interval between IAPS trials. TFUS targeting the amygdala decreased inter-beat intervals (i.e., increased heart rate) during inter-trial intervals of the mildly aversive IAPS task. Increased inter-trial intervals indicate a lower heart rate. A violin plot of data in the main manuscript in Figure 7.

Table S3A. Individual Arousal Ratings Aggregated by Session Trials

	REACTIVITY						REAPPRAISAL					
	Amygdala			Entorhinal Cortex			Amygdala			Entorhinal Cortex		
	Pre	Post	Difference	Pre	Post	Difference	Pre	Post	Difference	Pre	Post	Difference
	7.42	4.88	-2.54	6.42	1.25	-5.17	-0.42	0.08	0.50	-1.25	0.10	1.35
	0.63	1.79	1.16	-0.08	0.33	0.41	-0.04	-0.02	0.02	-0.08	-0.02	0.06
	4.89	5.96	1.07	6.36	7.33	0.97	0.12	-0.46	-0.58	-0.27	-0.06	0.21
	2.04	4.50	2.46	-2.67	-3.83	-1.16	-1.31	-3.58	-2.27	1.33	3.29	1.96
	1.83	2.33	0.50	1.92	3.29	1.37	0.08	-0.10	-0.18	-0.40	-0.33	0.07
	1.79	0.46	-1.33	0.96	0.46	-0.50	-0.10	-0.04	0.06	-0.13	0.27	0.40
	3.83	6.71	2.88	2.17	1.83	-0.34	0.19	0.04	-0.15	-0.17	0.00	0.17
	-3.00	-2.96	0.04	0.00	0.00	0.00	0.29	-0.13	-0.42	0.00	0.00	0.00
	2.46	2.38	-0.08	5.50	6.21	0.71	-0.21	-0.17	0.04	-0.15	-0.08	0.07
	0.46	1.46	1.00	1.63	1.75	0.12	0.27	-0.27	-0.54	0.06	-0.10	-0.16
	5.21	5.83	0.62	3.93	6.50	2.57	-0.69	-0.58	0.11	-0.50	-0.06	0.44
	1.31	1.04	-0.27	1.46	0.96	-0.50	-0.19	0.13	0.32	0.13	-0.10	-0.23
	-1.33	0.42	1.75	0.79	-0.25	-1.04	-0.79	-0.83	-0.04	-0.35	-0.33	0.02
	3.67	5.00	1.33	4.88	3.92	-0.96	0.02	0.00	-0.02	-0.38	-0.60	-0.22
	2.04	2.50	0.46	2.38	2.79	0.41	0.21	0.04	-0.17	0.19	-0.23	-0.42
	4.50	0.00	-4.50	4.54	6.00	1.46	0.67	0.00	-0.67	-0.35	-0.77	-0.42
	-3.17	0.00	0.28	-2.10	-0.48	-0.10	0.13	0.31	0.18	0.43	1.56	0.21
**Mean**	1.65	2.14	0.28	2.22	2.27	-0.10	-0.08	-0.27	-0.22	-0.10	0.10	0.21
**Min**	-3.17	-2.96	-4.50	-2.67	-3.83	-5.17	-1.31	-3.58	-2.27	-1.25	-0.77	-0.42
**Max**	7.42	6.71	2.88	6.42	7.33	2.57	0.67	0.31	0.50	1.33	3.29	1.96
**St Dev**	±2.55	±2.65	±1.68	±2.58	±3.08	±1.02	±0.48	±0.91	±0.32	±0.44	±0.97	±0.55

Table S3A. Individual Participant Arousal Ratings Aggregated by Session Trials
Table rows are in a randomized order; each row across in each table represents a unique participant's visits, with the order of visits also randomized.

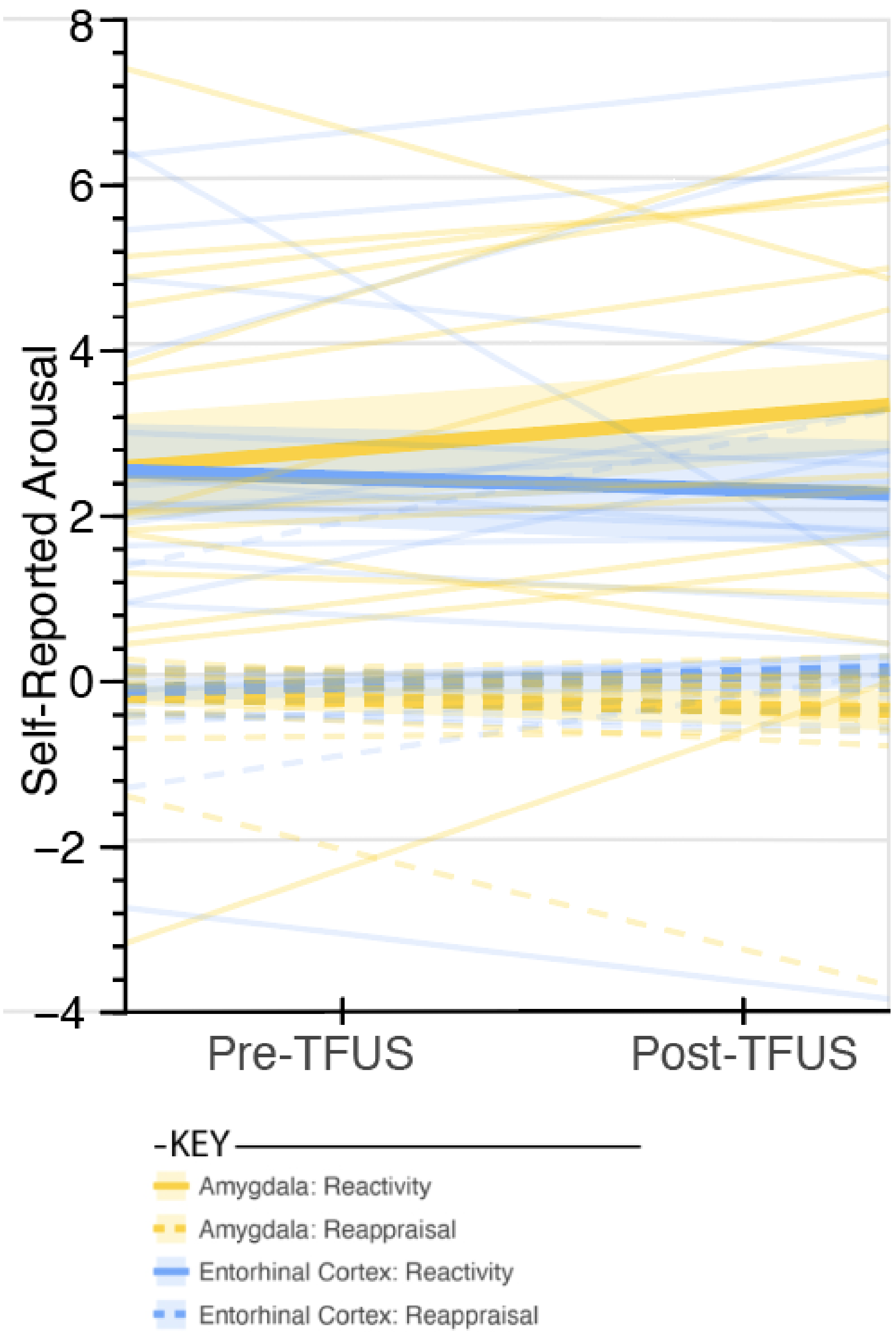

Figure S5A. Individual Participant Arousal Ratings Aggregated by Session Trials
Individual points are available in table Supplemental Table S3A, and a graph with the individual points and averaged findings are included in the main manuscript as Figure 5.

Table S3B. Individual Valence Ratings Aggregated by Session Trials

	REACTIVITY						REAPPRAISAL					
	Amygdala			Entorhinal Cortex			Amygdala			Entorhinal Cortex		
	Pre	Post	Difference	Pre	Post	Difference	Pre	Post	Difference	Pre	Post	Difference
	-4.33	-4.63	-0.30	-5.58	-5.46	0.12	0.29	0.13	-0.16	0.50	0.21	-0.29
	-2.00	-1.63	0.37	-1.88	-2.00	-0.12	0.04	0.10	0.06	-0.17	0.19	0.36
	-6.33	-6.13	0.20	-6.33	-7.58	-1.25	0.13	0.25	0.12	0.20	0.02	-0.18
	-4.21	-4.75	-0.54	-3.71	-4.00	-0.29	3.35	3.29	-0.06	0.46	-0.29	-0.75
	-2.29	-2.63	-0.34	-2.38	-3.46	-1.08	-0.21	0.19	0.40	-0.02	0.33	0.35
	-2.04	-2.13	-0.09	-1.08	-0.71	0.37	0.25	0.08	-0.17	0.04	0.04	0.00
	-4.00	-3.67	0.33	-3.17	-2.54	0.63	0.17	0.00	-0.17	-0.02	0.00	0.02
	-3.08	-3.38	-0.30	0.00	0.00	0.00	-0.44	-0.02	0.42	0.00	0.00	0.00
	-2.83	-1.79	1.04	6.17	6.88	0.71	0.48	-0.06	-0.54	-0.25	-0.19	0.06
	-2.92	-2.25	0.67	-1.71	-2.50	-0.79	0.00	0.06	0.06	0.08	0.19	0.11
	-5.04	-4.67	0.37	-3.53	-4.67	-1.14	0.29	-0.04	-0.33	0.50	-0.10	-0.60
	-1.81	-1.58	0.23	-2.00	-1.83	0.17	0.31	0.06	-0.25	-0.08	-0.08	0.00
	-2.88	-1.38	1.50	-3.38	-1.79	1.59	0.67	0.23	-0.44	0.94	0.13	-0.81
	-4.33	-6.13	-1.80	-4.54	-4.25	0.29	-0.31	0.19	0.50	-0.25	0.33	0.58
	-2.33	-3.63	-1.30	-2.42	-2.92	-0.50	-0.15	0.13	0.28	-0.08	-0.04	0.04
	-2.67	0.00	2.67	-3.42	-4.00	-0.58	-0.50	0.00	0.50	-0.17	0.17	0.34
	-4.17	-1.17	0.17	-4.29	-3.00	-0.12	0.25	0.19	-0.06	0.28	0.31	-0.05
**Mean**	-2.73	-2.55	0.17	-2.54	-2.58	-0.12	0.22	0.24	0.01	0.11	0.08	-0.05
**Min**	-6.33	-6.13	-1.80	-6.33	-7.58	-1.25	-0.50	-0.06	-0.54	-0.25	-0.29	-0.81
**Max**	-1.81	0.00	2.67	6.17	6.88	1.59	3.35	3.29	0.50	0.94	0.33	0.58
**St Dev**	±1.26	±1.79	±1.04	±2.72	±3.03	±0.76	±0.88	±0.81	±0.32	±0.32	±0.18	±0.39

Supplementary Table S3B. Individual Participant Valence Ratings Aggregated by Session Trials
Table rows are in a randomized order; each row across in each table represents a unique participant's visits, with the order of visits also randomized.

7. Discussion

No auditory or tactile sensations reported in either tFUS administration. Additionally, in our corresponding neuroimaging manuscript (Kuhn et al. 2023), no significant effect in the auditory cortex (all FDR-corrected p-values > 0.05).

Works Cited

1. Nasreddine ZS, Phillips NA, Bédirian V, Charbonneau S, Whitehead V, Collin I, *et al.* (2005): The Montreal Cognitive Assessment, MoCA: a brief screening tool for mild cognitive impairment. *J Am Geriatr Soc* 53: 695–699.
2. Bookheimer SY, Salat DH, Terpstra M, Ances BM, Barch DM, Buckner RL, *et al.* (2019): The Lifespan Human Connectome Project in Aging: An overview. *Neuroimage* 185: 335–348.
3. Ocklenburg S, Peterburs J, Mundorf A (2022): Hemispheric asymmetries in the amygdala: A comparative primer. *Prog Neurobiol* 214: 102283.
4. Simic G, Bexheti S, Kelovic Z, Kos M, Grbic K, Hof PR, Kostovic I (2005): Hemispheric asymmetry, modular variability and age-related changes in the human entorhinal cortex. *Neuroscience* 130: 911–925.
